# Evaluation of Cytokines in Endocervical Secretion and Vaginal pH from Women with Bacterial Vaginosis or Human Papillomavirus

**DOI:** 10.5402/2012/342075

**Published:** 2012-03-22

**Authors:** Ana Claudia Camargo Campos, Eddie Fernando Candido Murta, Márcia Antoniazi Michelin, Cleomenes Reis

**Affiliations:** ^1^Department of Microbiology, Institute of Tropical Pathology and Public Health, Federal University of Goiás, 74001-970 Goiâna, GO, Brazil; ^2^Discipline of Gynecology and Obstetrics, Oncological Research Institute (IPON), Federal University of Triângulo Mineiro, 38025-440 Uberaba, MG, Brazil; ^3^Discipline of Immunology, Oncological Research Institute (IPON), Federal University of Triângulo Mineiro, 38025-440 Uberaba, MG, Brazil

## Abstract

*Objective.* To verify the relationship between vaginal pH and human papillomavirus (HPV) infection and to measure cytokine levels in endocervical secretions of women with bacterial vaginosis (BV) or HPV. *Methods.* 173 women (16–48 years old) were enrolled and divided into groups: BV, HPV, and controls. Microbiological culture and vaginal pH were measured. HPV detect by PCR, and cytokines by ELISA (IL-2, IL-6, IL-10, IL-12, TNF-*α*, and IFN-*γ* cytokines). *Results.* Of 173 women, 60 were control group (34.7%) and 113 were distributed in HPV (*n=36*, 20.8%), BV (*n=36*, 20.8%), vaginitis (*n=30*, 17.3%) and, BV and HPV-associated groups (*n=11*, 6.4%). Vaginal pH > 4.5 was related with HPV infection. IL-2 and IL-12 were increased in BV and HPV groups, and IL-6 (only BV group), compared to control group. IL-12 and IFN-*γ* were higher in HPV than BV group. *Conclusion.* The increase of vaginal pH was associated with HPV infection; BV and HPV groups had a Th1 cytokines immune response.

## 1. Introduction

The vagina and cervix are the first lines of physical and immunological defense against sexually transmitted pathogens [1, 2]. The presence of potentially pathogenic microorganisms, including protozoans, yeast, bacteria, and viruses, initiate the immune response and result in increased vaginal secretion of immune stimulating molecules, irritation, vulvar pruritus, and a fetid odor, although in some situations it may be asymptomatic or oligosymptomatic [1, 3].

Bacterial vaginosis (BV) of undefined etiology is an inflammatory and recurring syndrome of the lower genital tract and is considered the most prevalent vaginal imbalance affecting women of reproductive age [4, 5]. BV is characterized by an alteration in the normal vaginal flora, in which the predominant lactobacilli are replaced by various other microorganisms. Increases in species such as *Gardnerella vaginalis*, *Mobiluncus *spp., *Bacteroides *sp., *Prevotella *spp., *Peptostreptococcus *spp., *Mycoplasma hominis*, or *Ureaplasma urealyticum *are routinely detected and are accompanied by a subsequent increase in the vaginal pH [5, 6]. Bacterially produced proteases degrade the mucus secreted from the cervix to facilitate the pathogen's ability to contact and breach the protective epithelial barrier [2].

The human papillomavirus (HPV) is a sexually transmitted DNA virus and is the main causative agent of cervical tumors [7]. Distinct profiles of microorganisms colonizing the vagina have been associated with the development of cervical intraepithelial neoplasia (CIN) and HPV infection [8–10]. HPV prevalence has been estimated at 27.4% worldwide, and the frequency of women infected by at least one of the oncogenic types has been shown to be significantly higher than that with only low-risk types (18.7% versus 7.5%) [11].

The physiological defense mechanisms targeting foreign microorganisms, such as viruses or bacteria, are mediated by the innate and adaptive immune responses [12]. The effector phases of both innate and adaptive immunity are often regulated by peptide molecules known as cytokines [2]. In innate immunity effector cytokines are produced by mononuclear phagocytes and epithelial cells, while in adaptive immunity they are produced by activated T lymphocytes that are capable of inducing inflammatory reactions [13, 14]. Many of these cytokines, known as interleukins (IL), are produced by certain populations of leukocytes residing in the bloodstream, including monocytes, neutrophils, or eosinophils [12, 15]. The pattern of immune response can be divided into Th1, Th2, and Treg, these three patterns are best known; however, others as the Th-17 have been described [16]. Increase in the levels of IL-10 from T lymphocytes in culture supports the hypothesis that the immunosuppression of Th2 cytokines may promote the development of cervical lesions [17].

Understanding the mechanisms mediating the infectious and inflammatory processes that occur in the vaginal region will contribute to our understanding of the pathogenesis of these infections. We chose to examine the localized cytokines IL-2, IL-12, TNF-*α*, and IFN-*γ* because they are representative of a type 1 T-helper lymphocyte (Th1) response that regulates cytotoxic T lymphocytes, which are in part responsible for clearing infections in the genital tract, in association with phagocytic cells and soluble antimicrobial factors. Additionally, the cytokines IL-6 and IL-10 were chosen because IL-6 function is an anti-inflammatory molecule and IL-10 is a general regulatory cytokine. According to previous studies there is a relationship between that virus and increased vaginal pH [6]. Therefore, our objectives are to verify the relationship between vaginal pH and HPV infection and to measure cytokine levels in endocervical secretions of women with BV or HPV.

## 2. Materials and Methods

### 2.1. Subjects

After approval by the Committee of Ethics for Human and Animal Research of the Goiás Anticancer Association (ACCG), the work was initiated. From April 2007 to February 2008, a total of 173 women between the ages of 16 and 48 years were prospectively enrolled; this age range was chosen because it usually corresponds with the more sexually active years for women. Initially, study participants were recruited from the Gynecology and Breast Service of the ACCG, but our enrollment was extended to the Basic Health Units located in distinct regions of the city of Goiânia, GO, Brazil.

The necessary sample size for this particular age group of women was estimated by considering the known prevalence of bacterial vaginosis (25%) [5] and vaginal HPV infection (15%) [5, 8, 18]. Based on this calculation and desired test power >80%, the study required 157 women; we further took into account a 10% potential loss to followup and determined that a total of 173 participants should be enrolled in the research study. The women were divided into BV and HPV groups and healthy controls. The control group will be selected according to Gram staining showing exclusive presence of lactobacilli, along with negative cultures for other microorganisms and clinical features compatible with normality.

### 2.2. Sample Collection and Preparation for Analysis

After each participant gave written informed consent, biological material was collected from the posterior fornix by using a gynecological brush [17]. The sample was immediately inoculated onto Columbia-CNA (colistin—nalidixic acid) agar with 5% human blood and into tubes with BHI-PRAS broth that had been prereduced, anaerobically sterilized, and supplemented with hemin, vitamin K, and yeast extract. The plates and tubes were immediately placed in an anaerobic jar and transported to the laboratory for processing as described [8]. We also measured the individual's vaginal pH (posterior cul-de-sac), performed an amine test [5], and prepared histological slides for a Pap smear and a Gram stain.

Diagnosis of bacterial vaginosis was made when three of the four established clinical criteria were met: vaginal discharge, pH > 4.5, positive amine test, and the presence of clue cells [4]. Presumptive identification was made when typical bacterial small colony morphology was observed and beta hemolysis was detected upon incubation at 35°C for up to 72 hours in an atmosphere of 5–7% CO_2_. In those cases, diagnosis was confirmed by tests of carbohydrate fermentation, motility, and rapid hippurate hydrolysis [17].

The sample that had been collected from the endocervix was used for laboratory measurement of cytokine levels and the presence of high-risk and low-risk HPV. The tube containing the endocervical secretion was centrifuged and saline (0.85%) was added to obtain a final volume of 500 *μ*L, which was then aliquoted and stored at −70°C until use for cytokine measurement by ELISA. The cultured plate was used for microbiological analysis after 48–72 hours of incubation at 37.0 ± 0.5°C.

### 2.3. Cytokine Detection by Enzyme-Linked Immunosorbent Assay (ELISA)

For cytokine measurement, an aliquot of the endocervical secretion was thawed and subjected to ELISA using pairs of commercially available monoclonal antibodies (BD Biosciences, Franklin Lakes, NJ, USA). All cytokines were standardized in a concentration of 10 ng/mL, except for that for TNF-*α* which was standardized at 100 ng/mL. The ELISAs for the samples and controls were performed in duplicate at the Oncology Research Institute of Federal University of Triângulo Mineiro—UFTM. Standard recombinant cytokines were used as controls. Samples previously diluted in saline were further serially diluted with assay diluent (PBS and fetal bovine serum), according to the BD recommendations. The spectrophotometric absorbance at 405 and 490 nm was measured and the difference of the values was multiplied by the dilution factor of the initial dilution of the samples in saline. The concentration of cytokines was determined in pg/mL by comparing the absorbances with those of a standard curve for the respective recombinant cytokine [19].

### 2.4. DNA Extraction and Polymerase Chain Reaction (PCR)

We diluted 100 *μ*L of the initial 500 *μ*L sample in TRIzol to extract DNA, according to the manufacturer's instructions (Invitrogen, Carlsbad, CA, USA). We performed PCR to test for the presence of low-risk HPV 6 and 11 (40 cycles) and high-risk HPV 16, 18, 31, 33, and 35 (35 cycles) using the primers 5′ to 3′ TACACTGCTGGACAACATGC and 5′ to 3′ GTGCGCAGATGGGACACAC, for the low-risk strains, and 5′ to 3′ TTTGTTACTGTGGTAGATACTAC and 5′ to 3′ GAAAAATAAACTGTAAATCATATTC for the high-risk strains, as previously described [18, 20]. The amplified products were mixed with 5 *μ*L of sample buffer and electrophoresed through a polyacrylamide gel (10%) at 90 volts for 2 hours and stained with 2% silver nitrate. A standard 50 bp molecular weight marker was used on each gel (Trackit 50 bp DNA ladder, Invitrogen). The gel was then analyzed for the presence of specific banding patterns [21].

### 2.5. Statistical Analysis

Descriptive statistical analyses were carried out, including examination of the frequency distributions of categorical data. The prevalence was calculated to reflect the relative frequency of genital infections using confidence intervals (CIs) and *odds ratios*. The chi-squared and Mann-Whitney tests were used to determine if differences among the groups reached statistical significance (*P* < 0.05).

## 3. Results

The women were divided into three groups according to presence of BV, high- and low-risk HPV infection or noninfected healthy controls (see Table 1). Of a total of 173 women enrolled in the study, 60 (34.7%) belonged to the control group and 113 had the following division: HPV (*n* = 36, 20.8%), BV (*n* = 36, 20.8%), vaginitis (*n* = 30, 17.3%), and BV and HPV-associated were in (*n* = 11, 6.4%). Of the 47 patients infected with HPV, 36 were only by this virus and 11 were associated with BV. Among those infected only with HPV 19 (52.8%) had only high-risk HPV, 12 (33.3%) the low-risk, and 5 (13.9%) had both subtypes. Coincidentally, the same number of patients was diagnosed with BV (47) and 36 (76.6%) had only this disease, 6 (12.8%) were associated with high-risk HPV, 4 (8.5%) associated with HPV low-risk, and only one of them had both BV and viral types. In women with BV, 41 (87.2%) were associated with the presence of *Gardnerella vaginalis* and 6 with other anaerobic bacteria.

The diagnosis of BV was made in 47 cases when three of the four routine clinical criteria were detected. In particular, pap smear analysis revealed clue cells in 41 of the cases. However, Gram staining was positive in 95.8% of the 47 diagnosed BV patients.

The women were divided into groups based on their clinical and microbiological characteristics. The normal flora in the control group was found to consist of *Lactobacillus *sp. and other bacilli and Gram-positive cocci. The 47 BV-positive cases harbored *Gardnerella vaginalis* (GV; in 87.2%), *Prevotella *sp. (in 42.5%), and *Clostridium *sp. (in 14.9%), in addition to other anaerobes. Among the 41 cases of GV diagnosed by culture, 11 also had HPV (26.8%; *P* = 0.373), and six of these 11 were of high risk (*P* = 0.850), four were of HPV low-risk (*P* = 0.616), and one had both low and high-risk strains (*P* = 0.200). In the group without GV, we observed a similar rate of HPV infection (36 cases; *P* = 0.787). The same comparisons were made for the group with and without increased anaerobic bacterial flora. Forty-three (27.0%) of the women who presented with the prevalent of anaerobic had HPV, which was similar to those in which we did not identify any prevalence of anaerobes (28.6%) (*P* = 0.902). There was not observed an association between the presence of HPV and the presence of predominant anaerobic bacteria however, we identified a statistical association between vaginal pH > 4.5 and HPV infection (Table 1).

Levels of the cytokines IL-2, IL-6, IL-12, IFN-*γ*, and TNF-*α* were measured in the presence of BV and HPV-infected women, and they were compared with their corresponding concentrations for the healthy control group. Only IL-2, IL-6, IL-10, and IL-12 levels were statistically different from controls; however IL-10, TNF-*α*, and IFN-*γ* were not (Table 2). The association between HPV and BV occurred in 11 patients with IL-12 of 143.00 pg/mL, a statistically significant difference when compared to the control group 44,30 pg/mL, *P* = 0.04. The association between high- and low-risk HPV (*n* = 6), BV and low-risk HPV (*n* = 4), BV and high-risk HPV (*n* = 6), and VB and high-low risks HPV (*n* = 1) could not be statistically determined due to the relatively low numbers of patients. The other cytokine concentrations and the association between bacterial vaginosis (BV), HPV, high-risk HPV, and low-risk HPV are in Table 2.

## 4. Discussion

In this study, our cohort exhibited incidences of BV and HPV that were higher than those previously reported [5]. This finding may be due to the fact that our collection strategy included women presenting at the Basic Health Units, which are the first place women come with primary health care concerns and by patients of tertiary care services.

 Papanicolaou smears are a routine method used to identify the presence of clue cells associated with BV [8, 22]. We performed this type of diagnostic analysis, but found that the Gram stain method was much more sensitive for detecting BV [5, 23]. Furthermore, we identified *Gardnerella vaginalis* as the main microorganism associated with the appearance of BV in our study cohort. These particular results are consistent with other previous studies [3, 5, 24]. The fact that some patients do not have vaginal pH > 4.5 does not exclude them from the group with BV [4].

Changes in the vaginal flora, such as the appearance of clue cells, suggest that BV occurs with a significantly higher frequency in women with cervical cytological abnormalities. A higher frequency of HPV has also been observed in these patients and is presumed to be supported by the production of nitrosamines by anaerobic bacteria and subsequent stimulation of cytokine production [18]. However, in our study, we did not find an association between HPV infection and the presence of *Gardnerella vaginalis *(*P* = 0.890),* Prevotella *sp. (*P* = 0.150), or any other species of anaerobic bacteria (*P* = 0.840) in the vaginal mucosa and conclude that the predominance of anaerobic microorganisms in vaginal flora was not significantly associated with the appearance of HPV infections.

Considering that BV, cervicitis, and CIN have been associated in some studies, it would not be surprising if the patients with BV and CIN presented with similar profiles in their localized immune response [17]. In contrast, our patient cohort did not exhibit a significant association between the presence of HPV and bacterial vaginosis.

One of the most common recognized factors among women with BV is the elevation of the vaginal pH [5]. Murta et al. (2007) measured the vaginal and the endocervical pH using a digital pH meter and concluded that endocervical pH was 5, 4 and it was different from posterior vaginal fornix with pH =4.9 [25]. Accordingly, we found a statistically significant association between pH and HPV in this study, where women with a pH >4.5 in posterior vaginal fornix (mean = 5.4) were predisposed to the appearance of HPV infections (*P* = 0.031) [18, 26]. Moreover, we believe that increased vaginal pH may be common in infections due to HPV and independent of the presence of BV. Another study concluded that BV was more common among women with high-grade squamous intraepithelial lesions (SIL) than in women with no cytological abnormalities, but further studies are required to confirm this hypothesis [27]. Knowing the environment that favors the appearance of the viral infection is possible to understand the pathogenesis of this viral infection and seek alternative prophylaxis.

Sampling the endocervical secretion is a reliable, highly reproducible method to detect cytokine concentrations in the genital tract [2]. By this method, we observed increased concentration of IL-10 in the presence of genital HPV infections and particularly in cases of infections with only high-risk HPV. The levels of IL-12 were likewise significantly higher in patients with HPV and only HPV infections compared to control and only BV groups. Findings by other authors have indicated a correlation between the concentrations of IL-10 and IL-12 and previous cervical HPV infection or infection with other pathogenic agents, such as HIV, which can compromise the integrity of the epithelial barrier and lead to accumulation of serum proteins in the cervix [28]. However, a study by Gravitt and colleagues determined that this association of an increase in IL-12 only occurred when there was a coinfection with HIV and HPV [29].

The local production of cytokines in cervix is important for the regulation of immunity in the genital tract [27]. Researchers have reported that an increase in IL-12 was present in women with increased vaginal pH, a characteristic of women with BV compared to health women [16, 29], and we also found statistically significant values. In our study, we found that increased IL-12 was associated with BV patients co-infected with HPV strains (*P* = 0.039), but not those who have coinfection with high-risk HPV strains (*P* = 0.256).

The levels of IL-2 are not increased in patients with HPV, HIV, or other STDs, probably because this cytokine is produced very early in the immune process and rapidly degraded [13]. However, we observed a significant increase of this cytokine in the presence of BV, total HPV, and high-risk HPV, demonstrating that the collection was carried out effectively and dose obtained before a possible degradation of IL-2, and we could also identify an increase of this cytokine in association of BV and HPV infections compared to BV group. Cytokines of the Th1-type (IL-2, 6, 12) enhance cell-mediated immune responses, whereas cytokines of the Th2-type (IL-4, 5, 10) inhibit it [12]. Tang et al. (2008) find that IL-2 deficiency contributes to intraislet Treg cell dysfunction and progressive breakdown of peripheral self-tolerance in the nonobese diabetic (NOD) mouse and Il-2 was originally discovered as a T-cell growth factor and activator of cytotoxic lymphocytes in inflammatory settings such as microbial infection [30]. Studies indicate an increase in IL-2 as an indicator of effective immune response during activation and proliferation of lymphocytes and their reduction as an immunosuppression factor.

These cytokines IL-6 and IL-8 are probably associated with the development of cervical cell lesions [17, 31]. These same authors also reported significant increases in IL-6, IL-8, and IL-10 in women with BV. In our study, we observed a significant increase in the levels of IL-6 in women with BV, but not in association with BV and coinfection with HPV. We also observed a tendency to increase IL-10 levels in patients with HPV occurred simultaneously with other infections. Cytokines of the Th1-type (IL-2, 6, 12) could enhance cell-mediated immune responses, like those probably seen in BV [12]. The literature also provides evidence that increases in IL-10 occur in lymphocytic infiltrates that correspond to HPV infections and are related to the Th2-type response. Likewise, increasing pH is a characteristic of immune responses and elevation of pH is considered to be a predisposing factor for HPV infection [29].

In this study, the levels of IFN-*γ* in women with HPV except in low-risk HPV were rather above those of the control group. According to a previous study, the presence of HPV associated with the regression of cervical lesions may be the result of a Th1 response (decreased IL-8 and increased IFN-*γ*). Although one study reported a decrease of IFN-*γ* during HPV infections [17], our study show a significant increase of this cytokine in the presence of HPV infection or BV compared to health women, although when this cytokine was evaluated in only HPV infections or only BV group, its became lower than control group. We could relate that the isolated presence of these complications would decrease the Th1 response. This finding may be related to other factors that were not evaluated in our study, such as hemoglobin contamination, volume of secretion, or day of menstrual cycle [29].

The increases in TNF-*α* have not been reported as significantly different between BV-positive and -negative subjects [32]. Due to the instability of this cytokine in ambient temperatures, we measured it first, as suggested by Sturm-Ramirez et al. (2000) [4]. Previous studies have identified a localized increase in cytokines in the presence of anaerobic bacteria, such as *Gardnerella *sp. and *Prevotella *sp., having a Nugent score >4 when compared to a group with an abundance of *Lactobacillus* sp. [32]. An increase in TNF-*α* and leukocytes in BV-diagnosed women with GV has also been reported [4]. We need to point out, however, that we did not observe a relationship between high levels of TNF-*α* and the presence of BV or in association with BV and HPV infections. Further studies are needed to confirm the lack of change in TNF-*α* for cases of HPV infections and BV. Other vaginal infections also are influenced by the concentration of cytokines. In candidiasis, increases in IL-10 and decreases in IFN-*γ* have been reported [33]. Trichomoniasis appears to be associated with high levels of IL-10 and IL-12 [13]; however, that particular study was focused only on HPV infection in the presence of BV as reported in recent study [34].

Unfortunately, it wasnot possible to eliminate the natural variations in cytokine concentrations that occur during different phases of the menstrual cycle because the samples were collected at different times of the cycle, and it was difficult to accurately determine the duration of the infection for the individuals included in our study. Stages of infection can also influence the different cytokine levels and the secreted cytokine profile can influence the outcome of an infection. A cohort in order to control these factors could contribute for analyzing those variables.

We conclude elevation of vaginal pH (>4.5) was frequent in genital HPV infections. We also found IL-2 and IL-12 was increased in individuals with BV and HPV infections, whereas individuals with only BV had higher cytokine levels of IL-2, IL-12, and IL-6, compared to control group, the cytokines IL-12 and IFN-*γ* were higher in HPV group than only BV. By comparing the levels of cytokines in vaginal secretion among high-risk HPV, low-risk HPV, and BV groups, we found that a Th1 immunological response was occurring.

## Figures and Tables

**Table 1:** Association between BV, HPV, HR HPV, and LR HPV and vaginal pH >4.5.

	pH > 4.5		pH ≤ 4.5		OR	CI	*P*
	*N*	%	*N*	%			
Only HPV	23	63.9	13	36.1	2.25	1.066–1.887	0.031
Only BV	26	72.2	10	27.8	3.05	1.196–2.062	0.005
BV and HPV	11	100.0	0	0.0	24.76	1.770–2.437	<0.001
BV and HR HPV	6	100.0	0	0.0	13.16	1.727–2.344	0.029
BV and LR HPV	4	100.0	0	0.0	13.16	1.727–2.344	0.029
BV and HR and LR HPV	1	100.0	0	0.0	2.86	1.689–2.262	0.330
Control group	19	31.7	41	68.3	0.28	0.343–0.762	<0.001

BV: bacterial vaginosis, HPV: human papillomavirus, *n*: number of cases, OR: odds ratio, CI: confidence interval of 95%, HR: high risk, LR: low risk.

**Table 2:** Concentration of IL-2, IL-12, TNF-*α*, IFN-*γ*, IL-6, and IL-10 (median, 25–75 percentil in pg/mL), in women with bacterial vaginosis (BV), HPV, HR HPV, and LR HPV.

Cytokines	IL-2	IL-12	TNF-*α*	IFN-*γ*	IL-6	IL-10
Groups (*n*)						
HPV (47)	40.1 (24.3–77.8)^1^	92.3 (48.1–146.3)^6, 19^	40818 (20280–66630)	384.5 (269.3–644.4)^19^	979056 (384200–1191000)	142421 (79730–257000)^16^
HR HPV (31)	39.3 (23.6–85.7)^2^	92.3 (38.9–143.0)^19^	36090 (19520–62320)	375.0 (130.4–641.7)^11, 19^	892700 (139400–1170000)	149976 (85890–323300)^17^
LR HPV (22)	40.1 (24.0–91.7)	112.7 (53.9–177.9)^7, 19^	48170 (27120–76710)	407.0 (133.7–616.6)^19^	1038345 (573400–1223000)	131007 (68310–281100)
Only HPV (36)	36.3 (24.0–69.8)^3, 19^	90.5 (47.4–127.6)^19^	41680 (23080–66270)	4.6 (0.5–60.7)^12^	936400 (144600–1200000)	146100 (76670–276500)
Only HR HPV (19)	35.1 (24.1–58.1)^19^	91.9 (36.1–139.1)^19^	37030 (20280–66630)	2.8 (0.5–33.0)^12, 20^	621600 (57060–1079000)^22^	176500 (79730–323300)
Only LR HPV (12)	50.5 (24.0–69.8)^19^	82.8 (47.8–190.0)^19^	41680 (27410–66790)	50.8 (0.5–157.5)^13^	1038000 (567000–1200000)	131000 (68310–149800)
BV (47)	47.8 (21.9–90.6)^4^	104.1 (47.1–255.6)^8^	40996 (19520–71550)	421.6 (133.7–652.7)	976056 (403600–1316000)^14^	100763 (57490–224000)
Only BV (36)	47.8 (21.9–102.8)^5^	74.7 (44.4–261.8)^9^	41230 (17070–67460)	30.5 (6.2–70.4)	1099000 (346800–1413000)^15^	126400 (55910–235400)
BV and HPV (11)	55.4 (24.2–88.2)^19^	143.0 (53.7–272.3)^10, 19^	21820 (19520–75640)	30.4 (11.8.7–79.5)^18^	974300 (624100–1154000)	101700 (85890–257000)
BV and HR HPV (6)	61.9 (31.3–88.2)^21^	102.3 (29.4–291.3)^19^	20330 (15500–33070)	16.0 (7.0–34.8)^18^	1039000 (595600–1249000)	106000 (93100–187900)
Control group (60)	20.6 (5.4–67.4)	44.3 (27.7–113.1)	38530 (19270–84160)	367.0 (128.6–515.3)	979056 (384200–1191000)	88230 (47960–220500)

HR and LR HPV: 6; HPV: human papillomavirus, *n*: number of cases; *α* = 5%; HR: high-risk; LR: low-risk; *P*: significant difference between the concentration of cytokines compared to control group. ^1^0.022; ^2^0.047; ^3^0.040; ^4^0.009; ^5^0.012; ^6^0.020; ^7^0.029; ^8^0.006; ^9^0.012; ^10^0.039; ^11^0.053; ^12^<0.001; ^13^0.0012; ^14^0.034; ^15^0.019; ^16^0.061; ^17^0.054; ^18^<0.0001.

*P*: significant difference between the concentration of cytokines compared only BV to HPV infection: ^19^<0.0001; ^20^0.020; ^21^0.005; ^22^0.060.
